# Predictability modulates the anticipation and perception of pain in both self and others

**DOI:** 10.1093/scan/nsz047

**Published:** 2019-06-25

**Authors:** Weiwei Peng, Xiaoxuan Huang, Yang Liu, Fang Cui

**Affiliations:** 1 School of Psychology, Shenzhen University, Shenzhen, 518060, China; 2 Center for Brain Disorders and Cognitive Neuroscience, Shenzhen, 518060, China; 3 Shenzhen Key Laboratory of Affective and Social Cognitive Science, Shenzhen University, Shenzhen, 518060, China

**Keywords:** pain, predictability, self-pain, other-pain, event-related potentials, sensorimotor α-oscillation

## Abstract

Predictability has been suggested to modulate both the anticipation and perception of self-pain. Considering the overlapping neural circuits between self-pain and other-pain perceptions, the present study investigated how the predictability of forthcoming pain modulates the anticipation and perception of self-pain and other-pain. We used a balanced, within-participant experimental design in which a visual cue indicating the recipient, intensity and predictability of an upcoming painful electrical stimulation was presented before its delivery. Subjective ratings and electroencephalography activities to the anticipation and perception of self-pain and other-pain were recorded and compared between certain and uncertain conditions. Results showed that predictability affected the perception of self-pain and other-pain in a similar manner such that the differences in behavioral ratings and event-related potentials to high-intensity and low-intensity pain were significantly reduced when the intensity was uncertain. The strengths of predictability-induced modulation of self-pain and other-pain perceptions were positively correlated with each other. Furthermore, predictability also modulated the anticipation of both self-pain and other-pain such that pre-stimulus high-frequency α-oscillation power at sensorimotor electrodes contralateral to the stimulation side was maximally suppressed when anticipating certain high-intensity pain. These findings demonstrate that predictability-induced modulation on pain anticipation and perception was similarly applied to both self-pain and other-pain.

## Introduction

The ability to predict the likelihood of an aversive event reflects an adaptive capacity in humans, e.g. the certitude that pain will be forthcoming influences its perception (Ploghaus *et al.*, 2003; Koyama *et al.*, 2005). Knowing that pain will occur is thought to be associated with greater fear and hypoalgesia (Petrovic *et al.*, 2005; Labrenz *et al.*, 2016), which are triggered through the descending activation of pain-modulation systems including the rostral anterior cingulate cortex and periaqueductal gray (Ploghaus *et al*., 2003; Lin *et al*., 2014). When pain can be anticipated with certainty, selective attention can be more precisely directed to the upcoming sensations, and neural activities within brain regions that govern sensory-discriminative processing (e.g. primary and secondary somatosensory cortex) would increase, which leads to better discriminative processing of nociceptive stimuli (Carlsson *et al*., 2000, 2006). In contrast, in both humans and rats, when pain cannot be anticipated with certainty, there would be increased anxiety and hyperalgesia, along with greater physiological arousal (Oka *et al*., 2010; Schaap *et al*., 2013; Labrenz *et al*., 2016). When pain is unpredictable, individuals have greater anxiety, which exacerbates the subjective perception of pain intensity as well as neuronal responses to constant pain stimulation (Ploghaus *et al*., 2001). This is true especially within brain regions associated with the processing of the affective aspect of pain (Carlsson *et al*., 2006), e.g. the anterior insula.

It is of crucial implications for self-protection and survival to anticipate sensorimotor events (e.g. sensory stimulation or motor response) as an adaptive reaction to the environment. In addition to the process of pain experience, predictability also modulates the cortical processing of anticipating the forthcoming pain. For example, depending on the level of predictability, distinct cortical networks are involved in the anticipation of pain (Brown *et al*., 2008). Certain anticipation involves cortical areas associated with semantic and prospective memory (i.e. anterior prefrontal, inferior frontal and temporal cortices), while uncertain anticipation involves a cortical network associated with attention (i.e. prefrontal, posterior cingulate and bilateral inferior parietal cortices). More directly, potent uncertainty-induced hyperalgesia can be predicted by the uncertainty-related anticipatory brain responses in the periaqueductal gray that plays a complex role in pain inhibition and facilitation (Yoshida *et al*., 2013). These findings suggest that predictability (i.e. certain/uncertain) modulates brain responses to both pain anticipation and perception. Indeed, this could be supported by evidence that uncertainty-induced anxiety was shown to have a negative bias for detecting an upcoming threat (Mogg and Bradley, 1998; Shihata *et al*., 2017), thus leading to increased behavioral and neuronal responses to the upcoming stimulation.

The functional state of distributed cortical networks could be indexed by ongoing neuronal oscillations (Laufs, 2008; de Silva, 2013) as effectively measured by electroencephalography (EEG) or magnetoencephalography. The cortical processes preparing the elaboration of sensorimotor events could be reflected by the anticipatory electroencephalographic α-oscillations (Klimesch *et al*., 2007; Jensen and Mazaheri, 2010; Klimesch, 2012; Babiloni *et al*., 2014), including both low-frequency α-oscillation (8–11 Hz) indexing the general tonic alertness and high-frequency α-oscillation (11–14 Hz) indexing the regulation of task-specific sensorimotor processes. Compared with a non-painful event, anticipating an upcoming painful event would induce decreased high-frequency α-oscillation power over the primary sensorimotor cortex (Babiloni *et al*., 2003), but this effect was not observed for low-frequency α-oscillation. More directly, the pre-stimulus α-oscillation over contralateral sensorimotor cortex preceding the painful stimulation has been shown to influence the subsequent evaluation of pain intensity (Babiloni *et al*., 2006) and cortical responses to the painful stimulation (Babiloni *et al*., 2008; Peng and Tang, 2016; Tu *et al*., 2019), such that the lower the anticipatory α-oscillation power, the higher the subjective evaluation of pain intensity, as well as the greater the cortical responses to the painful stimulation. These studies suggested that the pre-stimulus α-oscillation over the contralateral sensorimotor area, particularly the high-frequency α-oscillation, could reflect the brain states in the anticipatory stage and thus predict subjective pain perception as well as cortical responses to the subsequent painful stimuli. Based on this understanding, it is likely that predictability could modulate the pain anticipation process, which would be represented by the pre-stimulus α-oscillation over the sensorimotor cortex prior to the onset of pain stimulation, thus further influencing the subsequent pain perception to some extent.

The neural circuits underlying the perception of one’s own pain (self-pain) and that of others (other-pain) have been shown to overlap (Singer *et al*., 2004; Lawrence *et al*., 2006; Thioux and Keysers, 2010; Rutgen *et al*., 2015a,b). Moreover, they can be similarly modulated by psychological and pharmacological factors. For instance, the perception of both self-pain and other-pain can be modulated by placebo. Placebo analgesia that reduces the perception of self-pain also leads to decreased levels of self-reported empathy and neuronal activity associated with pain-related brain regions when processing other’s pain (Rutgen *et al*., 2015a). Blocking placebo analgesia using an opioid antagonist returns the empathic responses to normal (Rutgen *et al*., 2015b). Physical painkillers (i.e. acetaminophen) have also been shown to reduce empathy to other-pain via the same pathways that allow them to reduce self-pain (Mischkowski *et al*., 2016). This evidence supports the idea that the perception of self-pain and other-pain might be subserved by the overlapped neurocomputational functions and that empathizing with other-pain is, to some extent, grounded in first-hand painful experiences (Lamm *et al*., 2007; Bernhardt and Singer, 2012; Rutgen *et al*., 2015a,b).

The understanding that (i) the predictability (i.e. uncertain/certain) of pain modulates the perception of self-pain, (ii) pre-stimulus sensorimotor α-oscillations can reflect the readiness of the brain to respond to the upcoming painful stimulus and (iii) self-pain and other-pain share overlapping neuronal circuits led us to wonder whether predictability can modulate the perception of other-pain as it applies to the perception of self-pain. In the present study, we investigated whether the predictability of forthcoming pain modulates the processing of self-pain and other-pain. We used a balanced within-participant experimental design in which a visual predictability cue indicating the recipient (the participant or someone else), intensity (low or high) and predictability (certain or uncertain) of the forthcoming painful stimulation. Each participant was paired with someone that they thought was another participant and both had electrodes attached to their left hands to deliver electrical stimulation. Subjective ratings and neuronal responses [event-related potentials (ERPs)] to the perceptions of pain, as well as pre-stimulus sensorimotor α-oscillations during pain anticipation, were comprehensively compared. Further, we determined the correlation of predictability-induced modulation between self-pain and other-pain conditions.

Based on the aforementioned evidence, we have the following three hypotheses. First, while empathic pain is partially grounded in self-pain perception, predictability should modulate the perception of self-pain and other-pain in a similar way. Second, the influence that predictability has on self-pain and other-pain perceptions should be positively correlated with each other at the between-participant level, such that individuals who are more sensitive to predictability-induced modulation of self-pain will also tend to exhibit a greater modulatory effect on other-pain. Third, because pre-stimulus α-oscillations at the sensorimotor cortex, particularly for high-frequency α-oscillation, could predict subjective perception and cortical responses to the subsequent painful stimuli, they should reflect the degree of predictability-induced modulation in the anticipation of both self-pain and other-pain.

## Materials and methods

### Participants

Forty-three healthy right-handed volunteers (23 females) aged 20.47 ± 0.21 years (mean ± SEM, range = 18–23 years) were recruited to participate in the experiment. None of them reported cardiovascular or neurological diseases, acute or chronic pain, psychiatric disorders or current use of any medication. All participants gave their written consents and were informed of their rights to discontinue their participation at any time. They were informed that the study aimed to examine how individuals perceive self-pain and other’s pain. The experiment was conducted in accordance with the Declaration of Helsinki and was approved by the local ethical committee.

### Stimulation and experimental design

**Fig. 1:**
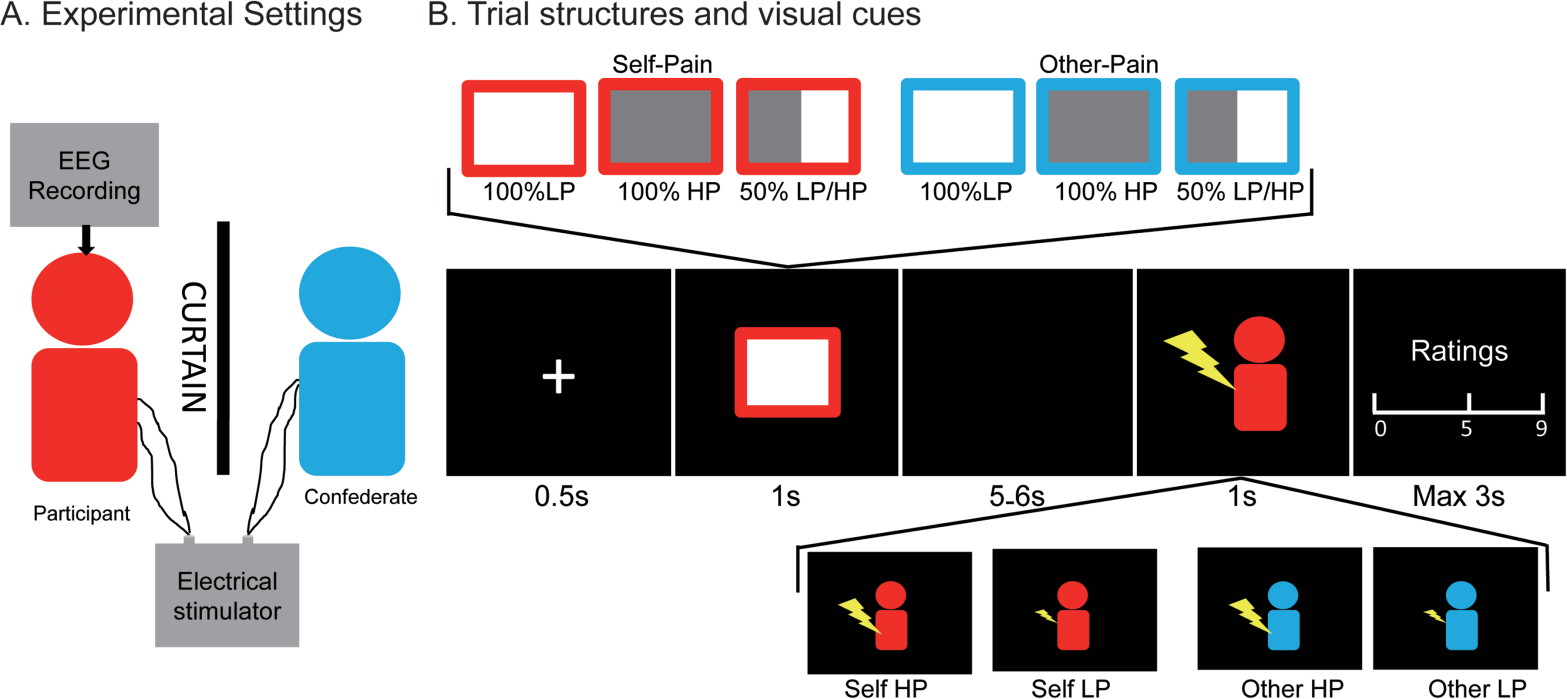
Experimental design. Participants either received electrical stimulation themselves or witnessed visual feedback indicating the delivery of electrical stimulation to another person (a confederate seated next to the participants in the EEG cabin, A). Each experimental trial started with a 0.5 s fixation, followed by the presentation of the visual predictability cue (duration: 1 s), which was completely predictive of the recipient and the intensity (100% LP, 100% HP or 50% LP/HP) of the upcoming electrical stimulation. This yielded six types of predictability cue (certain-LP, certain-HP, uncertain-LP/HP for self-pain and other-pain stimulaitons, respectively). After a blank screen (duration = 5–6 s randomly), the electrical stimulus (duration = 50 ms) was delivered, while another visual delivery cue (duration = 1 s) indicating the recipient (marked by the color of the cartoon) and intensity (marked by the size of the shock graphic) of the actual electrical stimulation was simultaneously shown on the screen. After each stimulation, participants were instructed to rate the intensity and unpleasantness of the pain on the 0–9 NRS.

We used a well-established empathy for pain paradigm (Singer *et al*., 2004, 2006), where participants received either low- or high-intensity electrical pain stimulations themselves or witnessed the low- or high-intensity electrical stimulations delivered to another person (who, in reality, was a confederate; Figure 1A). Electrical pain stimulation was square electric pulses with 50 ms duration delivered through ring electrodes attached to the fourth finger of the left hand. It was generated by a multichannel electrical stimulator (type: SXC-4A, Sanxia Technique Inc., China). Electrical pulse in either low intensity (1 mA, LP) or high intensity (4 mA, HP) was applied. We selected these two intensities based on a preliminary experiment, in which participants (*n* = 25) rated the perceived pain intensity at 1.53 ± 0.21 to the 1 mA electrical stimulation and at 6.06 ± 0.36 to the 4 mA electrical stimulation, on the 0–9 numerical rating scale (NRS) ranging from 0 (no pain) to 9 (unbearable pain). Furthermore, prior to the EEG recording, it was confirmed that each participant can well discriminate the low- and high-intensity electrical pain stimulations.

A visual cue indicating the recipient (to whom the forthcoming stimulation would be applied) and intensity of the upcoming electrical stimulation would be presented prior to the delivery of electrical stimulation, which could be referred to as ‘predictability cue’. The recipient of the upcoming electrical stimulation was either the participant self or the other participant (self-pain or other-pain), which was marked by the border color of the predictability cue. The intensity and predictability of the upcoming electrical stimulation intensity were marked by the proportion of the gray area in the predictability cue, including 100% of low-intensity pain (certain-LP), 100% of high-intensity pain (certain-HP) or 50% of low-/high-intensity pain (uncertain-LP/HP). It thus yielded six types of predictability cues (certain-LP, certain-HP and uncertain-LP/HP for self-pain and other-pain, respectively). It should be noted that the predictability was completely predictive to subsequent electrical pain stimulation (for both recipient and intensity), which would be explicitly informed to the participants. In addition, it would be confirmed that participants can correctly distinguish the visual cues prior to the EEG recording.

The experimental design was a 2 (recipient of electrical stimulation: self-pain *vs* other-pain) × 2 (predictability of upcoming electrical stimulation intensity: certain *vs* uncertain) × 2 (intensity of actually delivered electrical stimulation: LP *vs* HP) within-participant design. It thus resulted in eight conditions in total: certain-LP, certain-HP, uncertain-LP and uncertain-HP for self-pain and other-pain conditions, respectively. There were 40 trials for each condition (320 trials in total, 160 trials for self-pain and other-pain, respectively) presented in a pseudo-randomized sequence. As shown in Figure 1B, each experimental trial started with a 500 ms fixation, followed by the presentation of the predictability cue (duration = 1000 ms) indicating the recipient and intensity of the upcoming electrical stimulation. After a blank screen (duration = 5000–6000 ms randomly), the electrical stimulus (duration = 50 ms) was delivered, while another visual delivery cue (duration = 1000 ms) indicating the recipient and intensity of the delivered electrical stimulation (i.e. delivery cue) was simultaneously shown on the screen. The color of the cartoon and the size of the shock indicated the recipient (self or other) and intensity (LP *vs* HP) of actually delivered electrical stimulation, respectively. The presentation of the delivery cue was to enable participants to know how the electrical stimulation was delivered without directly witnessing other’s pain. After electrical stimulation of themselves (self-pain conditions), participants were instructed to rate the pain intensity and unpleasantness elicited by the electrical stimulation, on a 0–9 NRS ranging from 0 (no pain/unpleasantness) to 9 (unbearable pain/unpleasantness). After electrical stimulation of others (other-pain conditions), participants were instructed to rate the intensity of other’s pain and the unpleasantness in themselves, on the same 0–9 NRS (Figure 1B). It should be noted that the rating scale was presented visually on the screen, and participants would provide their ratings through the number pad on the keyboard, using their right hands.

### EEG recording

The participant was seated on a comfortable chair in a silent and temperature-controlled room (i.e. the EEG cabin), while the second participant (confederate) was seated next to him/her. Upon participants’ arrival to the laboratory, the real participant and the confederate (one of our experimenters) got to know each other briefly, and they would be informed that either of them would wear EEG caps in the experiment, which would be decided by drawing lots. Nevertheless, we would manipulate the outcome of drawing lots, such that only the real participant would be selected as the one to wear EEG caps. After the preparation of the experiment (e.g. wearing the EEG caps), the real participants and the confederate would be seated next to each other in the EEG cabin. Both of them would be attached with ring electrodes to the fourth finger of their left hands, through which the electrical stimulation would be delivered. They were told that at each experiment trial, either of them would receive electrical stimulation. Nevertheless, the confederate would not receive any actual stimulation during the experiment. To confirm that they cannot directly witness or hear each other during the experiment, both of them would wear noise-masking sleep buds and would be blocked by a curtain, as indicated by Figure 1A. Throughout the experiment, participants were instructed to focus on the stimuli and keep their eyes open. EEG data were collected using 64 Ag–AgCl scalp electrodes placed according to the international 10–20 system (Brain Products GmbH; passband: 0.01–100 Hz; sampling rate: 1000 Hz). The electrode FCz was used as the recording reference, and impedances of all electrodes were kept lower than 10 kΩ. Electro-oculographic (EOG) signals were simultaneously recorded using surface electrodes to monitor ocular movements and eye blinks.

### EEG data processing

EEG data were offline processed using EEGLAB (Delorme and Makeig, 2004), an open-source toolbox running in the MATLAB environment. Continuous EEG data were band-pass filtered between 0.2 and 30 Hz. EEG epochs were extracted using a window analysis time of 1500 ms (500 ms pre-stimulus and 1000 ms post-stimulus relative to the onset of electrical stimulation) and baseline corrected using the pre-stimulus interval. Trials contaminated by eye-blinks and movements were corrected using an independent component analysis (ICA) algorithm (Delorme and Makeig, 2004). In all datasets, these independent components had a large EOG channel contribution and a frontal scalp distribution. After ICA and additional baseline correction, EEG trials were re-referenced to the bilateral mastoid electrodes.

Epochs belonging to the same experimental condition were averaged and time-locked to the onset of the stimulus, yielding eight averaged waveforms for each participant and each electrode. We identified two main ERP peaks (N1 and P2) in the grand average waveforms that resulted from the self-directed electrical stimulation. N1 was defined as the most negative deflection 100–200 ms after stimulus onset with maximum distribution over the bilateral central region and P2 was the most positive deflection 200–400 ms after stimulus onset with maximum distribution at the central region. We identified three main ERP peaks (N1, P2 and P3) in the grand average waveforms that resulted from other-directed electrical stimulation. N1 was defined as the most negative component, 100–200 ms after stimulus onset with maximum distribution over the frontal-central area; P2 and P3 waves were defined as the most positive deflection, within the latency intervals of 200–300 and 300–800 ms, respectively, with maximum distribution over the central-parietal area. Amplitudes of these dominant waves were obtained from single-participant averaged ERP waveforms. Single-participant average waveforms were averaged to obtain group-level waveforms, and group-level scalp topographies at corresponding peak latencies were computed by spline interpolation. Based on the topographic distribution of grand average ERP activities and previous studies (Fan and Han, 2008; Peng *et al*., 2012; Liao *et al*., 2018; Hu and Iannetti, 2019), amplitudes of ERP components were measured at different sets of electrodes and latency intervals. In response to the self-pain, N1 and P2 amplitudes were measured at middle-central electrodes (C1, Cz and C2) within the latency interval of 90–120 and 200–230 ms, respectively. In response to the other-pain, N1 amplitudes were measured at frontal electrodes (F1, Fz and F2) within the latency interval of 130–160 ms and P2 and P3 amplitudes were measured at central-parietal electrodes (CP1, CPz and CP2) within the latency intervals of 190–220 and 350–500 ms, respectively.

### Pre-stimulus EEG spectral analysis

To assess how the predictability cue affected pre-stimulus neuronal oscillations during the anticipatory period (after cue presentation and before stimulation), pre-stimulus EEG signals were extracted using a 3000 ms time window (from −3000 to 0 ms relative to the stimulation onset). For each participant, pre-stimulus EEG signals for each of the six were transformed to the frequency domain using a discrete Fourier transform. This yielded six EEG spectra ranging from 1 to 30 Hz for each participant. Single-participant EEG spectra were subsequently averaged across participants to obtain group-level pre-stimulus EEG spectra for each cue type. Based on the understanding that (i) pre-stimulus α-oscillations over sensorimotor cortex can influence the subjective perception and cortical responses to subsequent sensory stimuli (Babiloni *et al*., 2006; Hu *et al*., 2013; Tu *et al*., 2016) and (ii) the neural functions of low- and high-frequency α-oscillations are different (Babiloni *et al*., 2014), we performed electrode-by-electrode two-way analysis of variance (ANOVA) with factors of ‘recipient’ (self-pain and other-pain) and ‘predictability’ (certain-LP, certain-HP and uncertain-LP/HP) on the pre-stimulus α-oscillation power within the low- (8–11 Hz) and high-frequency (11–14 Hz) α-bands separately. It thus yielded scalp distributions of *F* values and *P* values for each main effect and interaction effect, with dependent variables of low- and high-frequency pre-stimulus α-oscillation power, respectively. To account for the multiple comparison problems involved in the electrode-by-electrode analysis, the significance level was controlled using false discovery rate (FDR) (Benjamini and Hochberg, 1995; Genovese *et al*., 2002). Using this approach, the electrode cluster whose pre-stimulus α-oscillation power was significantly modulated by the main effect and/or the interaction effect was identified. Further, the obtained results of α-oscillation power were further confirmed using region-of-interest (ROI)-based statistical analyses.

**Fig. 2:**
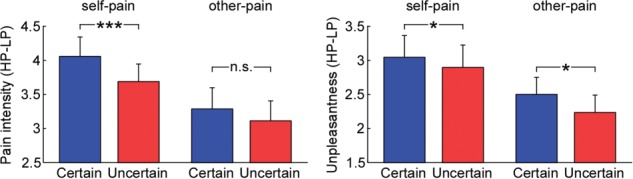
Influences of predictability on subjective ratings of pain intensity and unpleasantness. Differential ratings (HP − LP) of pain intensity and unpleasantness that were elicited by self-pain and other-pain stimulations were compared between certain and uncertain conditions. Data are mean ± SEM. Asterisks (^*^P < 0.05; ^***^P < 0.001; n.s.: *P* > 0.05) mark significance (pairwise *t*-test) for the hypothesis that uncertainty would reduce the perception of self-pain and other-pain.

### Statistical analysis

For data analysis, we used the statistics toolbox running under Matlab (The Mathworks, Natick, MA) as well as SPSS 20 (IBM Corp, Armonk, NY). For each participant and experimental condition, single-participant ratings of pain intensity and unpleasantness were calculated by averaging single-trial pain ratings across epochs belonging to the same experimental condition. Subjective ratings for pain intensity and unpleasantness were compared using a three-way repeated-measure ANOVA, with three within-participant factors of ‘recipient’ (self- and other-pain), ‘predictability’ (certain and uncertain) and ‘intensity’ (LP and HP). When the interaction among these three factors was significant, we performed a *post hoc* two-way ANOVA with factors of ‘predictability’ and ‘intensity’ for both self- and other-pain conditions. While self-pain and other-pain stimulations would elicit distinct ERP response patterns, particularly considering the different types of stimuli involved in these two conditions (sensory stimulation + visual delivery cue for self-pain condition, but visual delivery cue only for other-pain condition), we did not directly compare ERP amplitudes between self-pain and other-pain conditions. Instead, we performed separate two-way repeated-measure ANOVA with factors of ‘predictability’ and ‘intensity’ for the self-pain and other-pain conditions. When a main effect or interaction was significant, we performed *post hoc* pairwise comparisons.

**Table 1:** Predictability-induced modulation on subjective ratings (pain intensity and unpleasantness) and ERP amplitudes (N1 and P2 amplitudes elicited by self-pain; N1, P2 and P3 amplitudes elicited by other-pain)

	Certain		Uncertain		Two-way ANOVA		
	LP	HP	LP	HP	*F*_inter(1,42)_	*p*_inter_	${\eta}_p^2$
Self-pain condition							
Pain intensity	0.96 ± 0.18	5.01 ± 0.33	1.10 ± 0.20	4.78 ± 0.30	**16.29**	**<0.001**	**0.28**
Unpleasantness	0.93 ± 0.19	4.21 ± 0.38	1.00 ± 0.19	4.10 ± 0.35	**4.31**	**0.04**	**0.09**
N1 amp. (μV)	−4.05 ± 0.96	−9.65 ± 1.37	−5.13 ± 0.95	−9.13 ± 1.33	**4.47**	**0.04**	**0.10**
P2 amp. (μV)	14.12 ± 1.57	29.45 ± 1.93	14.74 ± 1.59	27.61 ± 1.86	**6.51**	**0.01**	**0.13**
Other-pain condition							
Pain intensity	1.14 ± 0.21	4.18 ± 0.39	1.21 ± 0.21	4.10 ± 0.39	2.72	0.11	0.06
Unpleasantness	1.04 ± 0.19	3.53 ± 0.32	1.16 ± 0.21	3.38 ± 0.30	**6.97**	**0.01**	**0.14**
N1 amp. (μV)	−1.43 ± 0.46	−0.24 ± 0.35	−0.61 ± 0.28	−0.74 ± 0.40	**5.70**	**0.02**	**0.12**
P2 amp. (μV)	1.47 ± 0.56	2.40 ± 0.47	2.21 ± 0.48	2.79 ± 0.55	0.51	0.48	0.01
P3 amp. (μV)	4.21 ± 0.60	6.00 ± 0.69	5.78 ± 0.60	6.15 ± 0.68	**4.44**	**0.04**	**0.10**

Note: Data are expressed using Mean ± SEM. Statistical results were obtained using two way repeated measure ANOVA with factors of `intensity’ (LP and HP) and `predictability’ (certain and uncertain). Statistics showing significant interactions are marked using bold.

## Results

### Behavioral results

A three-way repeated-measure ANOVA with factors ‘recipient’ (self-pain and other-pain), ‘predictability’ (certain and uncertain) and ‘intensity’ (LP and HP) was applied to the subjective intensity ratings. The analysis revealed a significant main effect of ‘intensity’ (*F*_(1,42)_ = 158.74, *P* < 0.001, ${\eta}_p^2$ = 0.79) in which pain intensity ratings to HP stimulation were significantly greater than those to LP stimulation. We also observed a significant interaction effect among these three factors (*F*_(1,42)_ = 12.63, *P* = 0.001, ${\eta}_p^2$ = 0.23). *Post hoc* two-way repeated-measure ANOVA with factors of ‘predictability’ and ‘intensity’ revealed the following results. (i) For self-pain conditions, we found a significant main effect of ‘intensity’ (*F*_(1,42)_ = 198.16, *P* < 0.001, ${\eta}_p^2$ = 0.83) and a significant interaction between the two factors (*F*_(1,42)_ = 16.29, *P* < 0.001, ${\eta}_p^2$ = 0.28). Pairwise comparisons showed that pain intensity ratings to uncertain-LP were significantly greater than those to certain-LP (*P* < 0.001), while ratings to uncertain-HP were significantly smaller than those to certain-HP (*P* = 0.001). The other direction of the pairwise comparison revealed that while pain intensity ratings to HP stimuli were significantly greater than those to LP stimuli for both certain and uncertain conditions (*P* < 0.001 for both comparisons), the differential ratings of pain intensity (HP − LP) were significantly greater on certain trials than those on uncertain trials (*P* < 0.001, left panel of Figure 2). (ii) For other-pain conditions, analysis only revealed a significant main effect of ‘intensity’ (*F*_(1,42)_ = 77.83, *P* < 0.001, ${\eta}_p^2$ = 0.65) in which pain intensity ratings to HP stimuli were significantly greater than those to LP stimuli. The interaction effect between ‘predictability’ and ‘intensity’ showed to be not significant (*F*_(1,42)_ = 2.72, *P* > 0.05, ${\eta}_p^2$ = 0.061), where the differential pain intensity ratings (HP − LP) did not differ between certain and uncertain conditions (*P* > 0.05, left panel of Figure 2). When directly applying pairwise comparisons between self-pain and other-pain conditions, subjective ratings of pain intensity to self-HP were significantly greater than those to other-HP (certain: *P* = 0.020; uncertain: *P* = 0.046), but no significant difference was observed for pain intensity ratings to self-LP and other-LP (*P* > 0.05 for both certain and uncertain conditions).

**Fig. 3:**
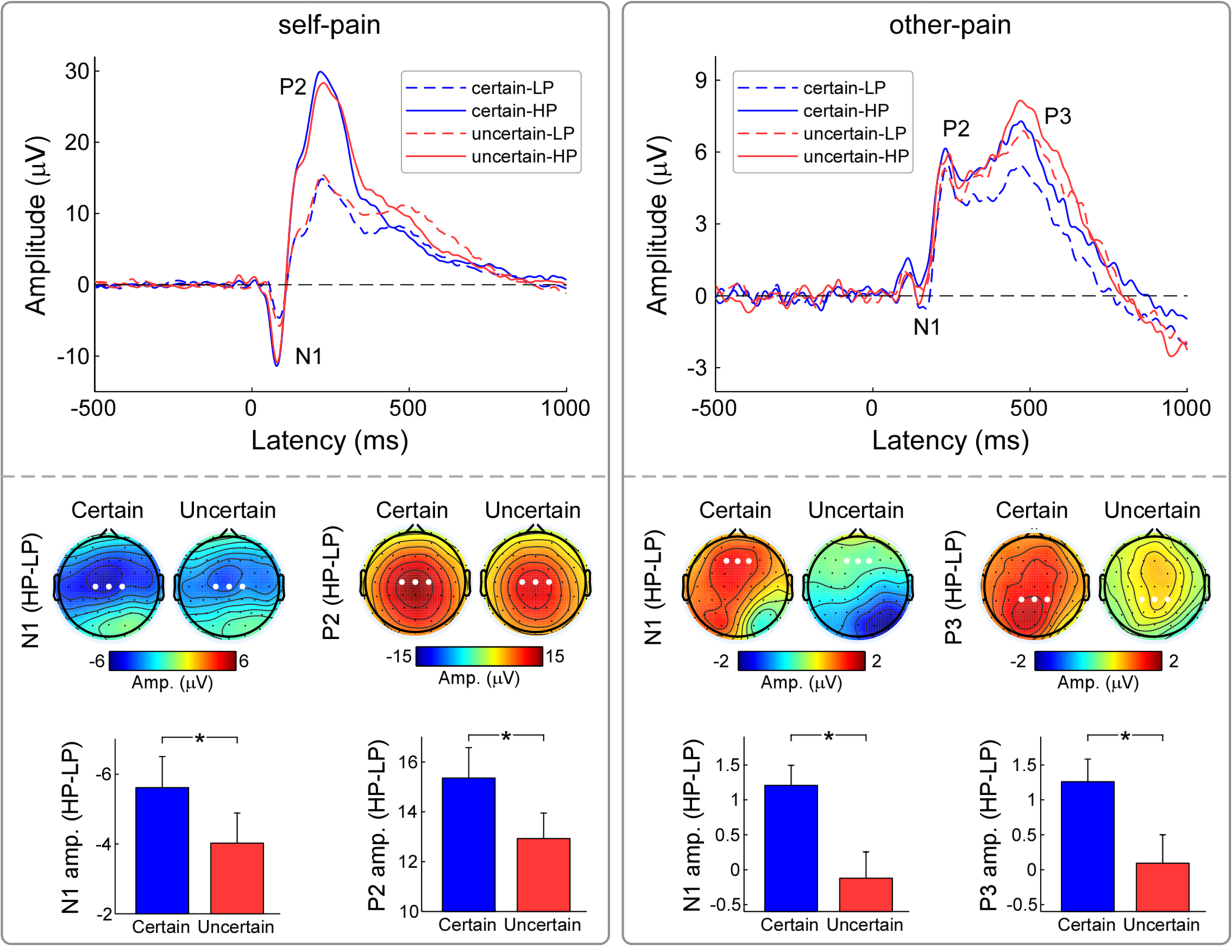
Influences of predictability on ERP responses to self-pain and other-pain. Group-level ERP waveforms (at Cz) were elicited by self-pain and other-pain in different conditions (certain-LP, certain-HP, uncertain-LP and uncertain-HP). Differential amplitudes (HP − LP) of ERP responses to self-pain and other-pain were compared between certain and uncertain conditions. Electrodes that are used to evaluate ERP amplitudes are marked using enlarged white dots in the corresponding scalp topographies. Data are expressed as mean ± SEM. Asterisks (^*^P < 0.05) mark significance (pairwise *t*-test).

The similar three-way repeated-measure ANOVA was applied to the subjective rating of unpleasantness. Analysis revealed a significant main effect of ‘recipient’ (*F*_(1,42)_ = 6.03, *P* = 0.018, ${\eta}_p^2$ = 0.13) in which unpleasantness ratings to self-pain stimulation were significantly greater than those to other-pain stimulation, as well as a significant main effect of ‘intensity’ (*F*_(1,42)_ = 106.93, *P* < 0.001, ${\eta}_p^2$ = 0.72) in which unpleasantness ratings to HP stimuli were significantly greater than those to LP stimuli. The analysis also showed a significant interaction between ‘recipient’ and ‘intensity’ (*F*_(1,42)_ = 16.26, *P* < 0.001, ${\eta}_p^2$ = 0.28). *Post hoc* pairwise comparisons revealed that while unpleasantness ratings to self-HP stimulation were significantly greater than those to other-HP stimulation (*P* = 0.001), ratings for self-LP and other-LP stimulation did not differ (*P* > 0.05). The analysis also showed that while unpleasantness ratings to HP stimuli were greater than those to LP stimuli for both self-pain and other-pain conditions (*P* < 0.001, for both comparisons), the differential unpleasantness rating (HP − LP) was significantly higher when stimulation was self-pain (*P* < 0.001).

Importantly, the analysis also revealed a significant interaction effect between ‘predictability’ and ‘intensity’ (*F*_(1,42)_ = 7.29, *P* = 0.010, ${\eta}_p^2$ = 0.15). *Post hoc* pairwise comparisons showed that unpleasantness ratings on uncertain-LP trials were significantly greater than those on certain-LP trials (*P* = 0.020), while ratings on uncertain-HP trials were significantly smaller than those on certain-HP trials (*P* = 0.031). The *post hoc* analysis also revealed that while unpleasantness ratings to HP stimuli were significantly greater than those to LP stimuli for both certain and uncertain conditions (*P* < 0.001 for both comparisons), the differential unpleasantness rating (HP − LP) was significantly greater when participants were certain about what stimulation would be delivered (*P* = 0.010, right panel of Figure 2). When directly applying pairwise comparisons between self-pain and other-pain conditions, subjective ratings of unpleasantness to self-HP were significantly greater than those to other-HP (certain: *P* = 0.005; uncertain: *P* = 0.002), but no significant difference was observed for unpleasantness ratings to self-LP and other-LP (*P* > 0.05 for both certain and uncertain conditions).

Across participants (*n* = 43), predictability-induced modulation of pain-intensity ratings on HP trials (estimated by HP_uncertain_ − HP_certain_), as well as that on LP trials (LP_uncertain_ −LP_certain_), correlated significantly between self- and other-pain conditions (HP: *r* = 0.742, *P* < 0.001; LP: *r* = 0.565, *P* < 0.001). Similar correlations were found for predictability-induced modulation of unpleasantness ratings (HP: *r* = 0.507, *P* < 0.001; LP: *r* = 0.476, *P* = 0.002). This pattern of correlations indicates a similarity between predictability-induced modulation of self-pain and other-pain at a between-participant level.

**Fig. 4:**
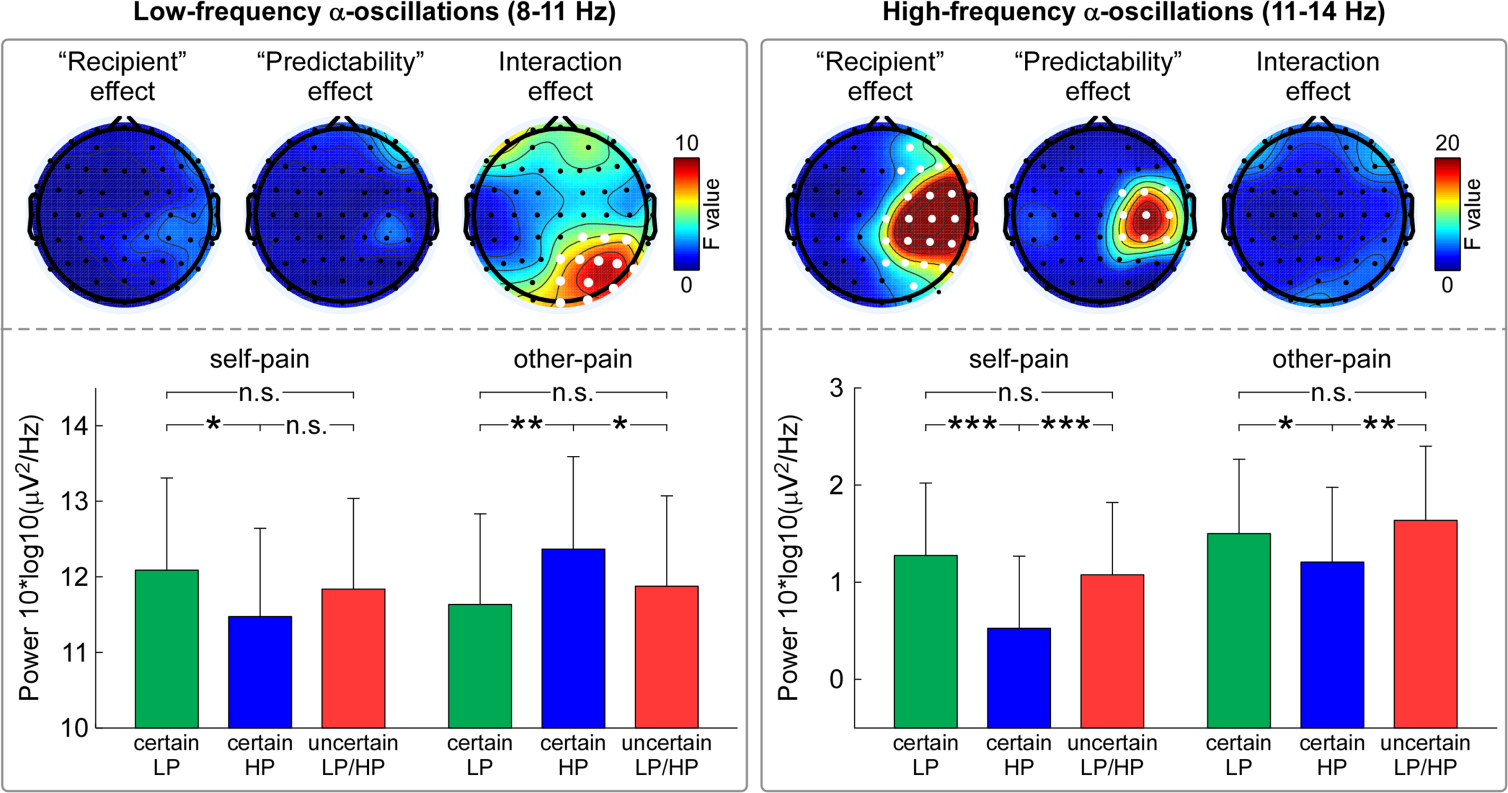
Influences of predictability on pre-stimulus α-oscillation power. Pre-stimulus α-oscillation power during the anticipation interval (after the presentation of the predictability cue and before the onset of electrical stimulation) was compared using repeated measure ANOVA with within-participant factor (‘recipient’ and ‘predictability’) for low-frequency (8–11 Hz, left panel) and high-frequency (11–14 Hz, right panel) α-band, respectively. For low-frequency pre-stimulus α-oscillation power, there was a significant ‘recipient’ × ‘predictability’ interaction at parietal-occipital electrodes contralateral to the electrical stimulation side (FDR-corrected *P* < 0.05, marked using enlarged white dots). For high-frequency pre-stimulus α-oscillation power, there were significant main effects of ‘recipient’ and ‘predictability’ at sensorimotor electrodes contralateral to the electrical stimulation side (FDR-corrected *P* < 0.05, marked using enlarged white dots). Note that pre-stimulus high-frequency sensorimotor α-oscillation power contralateral to the electrical stimulation side was significantly lower in the certain-HP condition than that in the certain-LP or uncertain-LP/HP conditions, regardless of self-pain or other-pain conditions. Data are expressed as mean ± SEM. Asterisks (^*^P < 0.05; ^**^P < 0.01; ^***^P < 0.001; n.s.: *P* > 0.05) mark significance (pairwise *t*-test).

### ERP results

The upper panel of Figure 3 is the group-level ERP waveforms (measured at the Cz electrode) elicited by the four self-directed and four other-directed conditions. Comparison of self-directed certain and uncertain conditions showed differential scalp topographies (HP − LP) for N1 amplitudes that were maximal at the central region contralateral to the stimulation side and those for P2 amplitudes that were more centrally distributed. For other-directed conditions, similar comparisons showed that differential scalp topographies for N1 amplitudes were maximal at the frontal region for certain trials and that those for P3 amplitudes were maximal at the central-parietal region.

As summarized in Table 1, a two-way repeated ANOVA with factors ‘predictability’ and ‘intensity’ was applied to N1 and P2 amplitudes that were elicited by self-pain stimulation. The analysis revealed significant main effects for ‘intensity’ (N1: *F*_(1,42)_ = 35.13, *P* < 0.001, ${\eta}_p^2$ = 0.46; P2: *F*_(1,42)_ = 171.50, *P* < 0.001, ${\eta}_p^2$ = 0.80) in which N1 and P2 amplitudes to self-HP stimuli were greater than those to self-LP stimuli. The analysis also revealed a significant interaction effect between the two factors (N1: *F*_(1,42)_ = 4.47, *P* = 0.040, ${\eta}_p^2$ = 0.10; P2: *F*_(1,42)_ = 6.51, *P* = 0.014, ${\eta}_p^2$ = 0.13). *Post hoc* pairwise comparisons showed that while both N1 and P2 amplitudes to self-HP were significantly greater than to self-LP (*P* < 0.001 for all comparisons) regardless of the level of certainty, the differential (HP − LP) N1 and P2 amplitudes were significantly greater when ‘predictability’ was certain than when it was uncertain (N1: *P* = 0.040; P2: *P* = 0.014, left panel of Figure 3). The other direction of the pairwise comparison revealed that (i) N1 amplitude to uncertain-LP was greater than that to certain-LP (*P* = 0.023), but did not differ between uncertain-HP and certain-HP (*P* > 0.05); (ii) P2 amplitude to certain-HP was greater than that to the uncertain-HP (*P* = 0.009), but no significant difference was observed between uncertain-LP and certain-LP (*P* > 0.05).

As summarized in Table 1, a two-way repeated-measure ANOVA with factors ‘predictability’ and ‘intensity’ was applied to N1, P2 and P3 amplitudes elicited by other-pain stimulation. The analysis revealed a significant main effect of ‘predictability’ (P2: *F*_(1,42)_ = 4.60, *P* = 0.038, ${\eta}_p^2$ = 0.10; P3: *F*_(1,42)_ = 5.52, *P* = 0.024, ${\eta}_p^2$ = 0.12), where other-pain stimulation elicited significantly greater P2 and P3 amplitudes when predictability was uncertain than when it was certain and a main effect of ‘intensity’ (N1: *F*_(1,42)_ = 6.94, *P* = 0.012, ${\eta}_p^2$ = 0.14; P2: *F*_(1,42)_ = 14.21, *P* = 0.001, ${\eta}_p^2$ = 0.25; P3: *F*_(1,42)_ = 10.80, *P* = 0.002, ${\eta}_p^2$ = 0.21), where other-HP elicited more positive N1, P2 and P3 amplitudes than those to other-LP trials. Further, we found a significant interaction between ‘predictability’ and ‘intensity’ for N1 and P3 amplitudes (N1: *F*_(1,42)_ = 5.70, *P* = 0.022, ${\eta}_p^2$ = 0.12; P3: *F*_(1,42)_ = 4.44, *P* = 0.041, ${\eta}_p^2$ = 0.10). *Post hoc* pairwise comparisons revealed that while N1 and P3 amplitudes on certain-HP trials were more positive than those on certain-LP trials (*P* < 0.001 for both comparisons), they did not differ between uncertain-HP and uncertain-LP trials (*P* > 0.05 for both comparisons). The differential (HP − LP) N1 and P3 amplitudes were significantly greater when ‘predictability’ was certain than when it was uncertain (N1: *P* = 0.022; P3: *P* = 0.041, right panel of Figure 3). The other direction of the pairwise comparison revealed that (i) P3 amplitude to uncertain-LP was greater than certain-LP (*P* < 0.001), but no significant difference was observed between uncertain-HP and certain-HP (*P* > 0.05); (ii) no significant difference of N1 amplitude was observed between uncertain-LP and certain-LP conditions (*P* > 0.05), as well as between uncertain-HP and certain-HP conditions (*P* > 0.05).

### Pre-stimulus EEG results

As revealed by the electrode-by-electrode two-way ANOVA with factors of ‘recipient’ (self-pain and other-pain) and ‘predictability’ (certain-LP, certain-HP and uncertain-LP/HP) on the low-frequency α-oscillation power, we identified the electrode cluster located at the parietal region contralateral to the electrical pain stimulation side, whose pre-stimulus low-frequency α-oscillation power was significantly modulated by the interaction of ‘recipient’ and ‘predictability’ (FDR corrected, left panel of Figure 4). ROI-based analysis further revealed that their pre-stimulus low-frequency α-oscillation power was significantly modulated by the interaction of ‘recipient’ and ‘predictability’ (*F*_(2,41)_ = 8.88, *P* = 0.002, ${\eta}_p^2$ = 0.17). *Post hoc* comparisons revealed that (i) for the self-pain condition, the α-oscillation power to certain-HP cues was only significantly lower than that to certain-LP cues (*P* = 0.022, left panel of Figure 4); (ii) for the other-pain condition, the α-oscillation power to certain-HP cues was significantly greater than that to both certain-LP and uncertain-LP/HP cues (*P* = 0.006 and *P* = 0.026, respectively, left panel of Figure 4). As revealed by the electrode-by-electrode two-way ANOVA with factors of ‘recipient’ (self-pain and other-pain) and ‘predictability’ (certain-LP, certain-HP and uncertain-LP/HP) on the high-frequency α-oscillation power, we identified the electrode cluster located at sensorimotor electrodes contralateral to the side of electrical stimulation, whose pre-stimulus high-frequency α-oscillation power was significantly modulated by the main effects of both ‘recipient’ and ‘predictability’ (FDR corrected, right panel of Figure 4). ROI-based analysis showed that their high-frequency α-oscillation power was significantly modulated by both ‘recipient’ (*F*_(2,41)_ = 28.03, *P* < 0.001, ${\eta}_p^2$ = 0.40) and ‘predictability’ (*F*_(2,41)_ = 16.63, *P* < 0.001, ${\eta}_p^2$ = 0.28). Specifically, the high-frequency α-oscillation power at sensorimotor electrodes contralateral to the electrical stimulation side was significantly lower in the certain-HP condition than that in the certain-LP (self-pain: *P* < 0.001; other-pain: *P* = 0.040) and uncertain-LP/HP conditions (self-pain: *P* < 0.001; other-pain: *P* = 0.007), similarly applied to both self-pain and other-pain (right panel of Figure 4).

## Discussion

The present study investigated the impact of predictability on anticipation and perception of self- and other-pain. We have obtained three main findings corresponding to our prior hypotheses. First, predictability modulated the perception of both self- and other-pain, such that uncertainty reduced the difference between HP and LP, well reflected by both subjective reports and ERP responses. Second, the modulation of pain perception induced by predictability was in the same manner for self- and other-pain, and they were significantly, positively correlated with each other at a between-subject level. Third, predictability also influenced pre-stimulus α-oscillations measured during the anticipatory stage for both self-pain and other-pain similarly. These results provide evidence for predictability-induced modulations of the anticipation and perception of self-pain and other-pain in a similar manner.

### Predictability-induced modulation on pain perception

When pain stimulation was directed toward the participants themselves, subjective ratings of both pain intensity and unpleasantness were significantly modulated by predictability. Participants definitely rated HP stimulation as more painful and more unpleasant than LP stimulation, but the difference in these ratings between HP and LP conditions was significantly smaller when it was uncertain than when it was certain. Consistently, predictability modulated the stimulus-evoked ERP responses such that the difference between HP and LP conditions in both N1 and P2 amplitudes was significantly lower in uncertain condition, compared to certain condition. N1 responses to self-pain have been shown to be functionally relevant to the sensory-discrimination aspect of painful experiences (Iannetti *et al*., 2005), such as encoding the location, intensity and quality of pain. P2 responses to self-pain have been suggested to reflect the affective-motivational salience and behavioral relevance of the painful experience (Zhang *et al*., 2005; Perchet *et al*., 2008; Rutgen *et al*., 2015a,b). Therefore, our behavioral and electrophysiological results together demonstrate that predictability can modulate both the sensory and affective dimensions of self-pain perception.

When the stimulation was directed to others, predictability exerted a similar modulation on behavioral (self-unpleasantness) and electrophysiological responses (N1 and P3 responses to the delivery cues) to other-pain. As in self-pain, the difference between HP and LP conditions was smaller when it was uncertain than when it was certain in other-pain on both behavioral and neural levels. Previous studies have demonstrated that observing other-pain involves (i) an early automatic, bottom-up process, reflected by the N1 and P2 components corresponding to emotional contagion and the affective sharing process and (ii) a later top-down controlled, cognitive process, reflected by the P3 component that regulates empathic responses (Fan and Han, 2008; Mella *et al*., 2012; Sessa *et al*., 2014). We observed N1, P2 and P3 responses elicited during other-pain perception. The reduced difference of N1 and P3 responses between LP and HP conditions in uncertain situations suggests that predictability modulates both the early affective-sharing stage as well as the later cognitive controlled stage. This result was consistent with a previous study showing that uncertainty-triggered anxiety exacerbated pain-related fear and arousal (Brown *et al*., 2008; Yoshida *et al*., 2013), whereas certainty induced better attention allocated to the upcoming stimulation, which led to better discrimination of sensory inputs (Carlsson *et al*., 2006).

Critically, predictability-induced modulation on other-pain perception shared very consistent patterns with that of self-pain perception, for both behavioral and neurophysiological responses. Specifically, the strength of the modulating effect on the behavioral response to self-pain (i.e. the difference of subjective self-pain intensity ratings and self-unpleasantness between certain and uncertain conditions) was significantly and positively correlated with that to other-pain (i.e. the difference of subjective other-pain intensity ratings and self-unpleasantness between certain and uncertain conditions). Particularly, ratings of unpleasantness presented the negative effect experienced by participants in response to self- or other-pain (Girard-Tremblay *et al*., 2014; Rutgen *et al*., 2015a,b). These findings demonstrate that the predictability-induced modulations might have shared underpinnings between self- and other-pain perception, at both within-participant and between-participant levels. Indeed, these findings further support the ideas that (i) empathy for other-pain and perception of self-pain recruit similar brain regions (Lamm *et al*., 2011) and that (ii) empathy for other-pain relies on a simulation of other’s feelings which are, at least partially, grounded in one’s own bodily, neuronal emotion systems (Gallese, 2008; Meng *et al*., 2013; Mier *et al*., 2014; Rutgen *et al*., 2015a,b).

### Predictability-induced modulation on pain anticipation

Inspired by the understanding that pre-stimulus sensorimotor α-oscillations have been suggested to predict subsequent pain perception (Zhang and Ding, 2010; Tu *et al*., 2016), we further investigated how predictability influenced pain anticipation by comparing the pre-stimulus α-oscillations after the presentation of different visual predictability cues (certain LP, certain HP and uncertain LP/HP). Results showed that predictability-induced modulation on the pre-stimulus high-frequency α-oscillation, measured at sensorimotor cortex contralateral to the electrical stimulation side, was similarly applied to the anticipation of both self-pain and other-pain, but this effect was not observed for low-frequency α-oscillations. Specifically, when anticipating certain-HP directed either to self or others, participants exhibited more suppressed sensorimotor high-frequency α-oscillation, as relative to certain-LP and uncertain-LP/HP conditions. Functionally, anticipatory α-oscillations at sensorimotor cortex have been suggested to reflect the cortical processes preparing the elaboration of sensorimotor events (Klimesch *et al*., 2007; Jensen and Mazaheri, 2010; Klimesch, 2012; Babiloni *et al*., 2014), where the low- and high-frequency α-oscillations reflect different neural functions. Low-frequency α-oscillation is suggested to reflect the general tonic alertness, while high-frequency α-oscillation is suggested to index the regulation of task-specific sensorimotor processes. Therefore, the similar predictability-induced modulation of pre-stimulus high-frequency α-oscillation when anticipating self-pain and other-pain could reflect the readiness of the neural system for the forthcoming pain, regardless of whether the forthcoming pain was directed to self or others. While sensory perception depends not only on the sensory inputs per se but also on the brain state prior stimulus onset (Kayser *et al*., 2016), it is likely that the different brain states that were captured by pre-stimulus sensorimotor α-oscillations would mediate the predictability-induced modulation on subsequent pain perception. On the other hand, this similar modulation of self-pain and other-pain anticipation also suggests that the mirror neuron system may provide a direct route to empathy for other-pain, including both the anticipation and perception stages. More specifically, the mirror neuron system that responds similarly to actions done by self and others (Kaplan and Iacoboni, 2006; Williams, 2008) may also involve in the anticipation of self-pain and other-pain.

### Limitations

There are several limitations that should be noted in the present study. First, the random interval (5000–6000 ms) between visual predictability cue and electrical stimulation onset may dilute the predictability-induced modulation effect on pain anticipation. It deserves to be further confirmed whether there would be a greater modulation effect on pre-stimulus α-oscillation if using a constant interval. Second, based on the spatial location of the observed predictability-induced modulation on pre-stimulus α-oscillations as well as the temporal sequence order of electrical stimulation onset and providing ratings, the observed modulation on pre-stimulus α-oscillation could be largely reflecting the cortical activities of anticipating forthcoming pain rather than preparing for ratings. However, the possible contribution of rating preparation on pre-stimulus α-oscillations could not be completely ruled out, since participants were required to provide their subjective ratings of pain intensity and unpleasantness to each electrical stimulation. Future studies could verify whether predictability can still influence pre-stimulus sensorimotor α-oscillations if participants only passively receive the pain stimulation without any instruction to provide pain ratings.

To sum up, the present study demonstrated that predictability influenced pain anticipation and perception, which was applied to self-pain and other-pain in a similar manner. This further expands the prior understanding for the overlapping between the perceptions of self-pain and other-pain based on similar psychological and neurocomputational functions.

## Funding

This study was funded by the National Natural Science Foundation of China (no. 31871127 to W.P. and 31871109 to F.C.), Shenzhen Basic Research Project (JCYJ20170818093231953 to W.P.) and Shenzhen University Natural Science Research Fund (2017073 to W.P.).

## Conflict of interest

None declared.
